# Synthesis of Mesoporous Metal Oxides by Structure Replication: Thermal Analysis of Metal Nitrates in Porous Carbon Matrices

**DOI:** 10.3390/nano5031431

**Published:** 2015-08-28

**Authors:** Christian Weinberger, Jan Roggenbuck, Jan Hanss, Michael Tiemann

**Affiliations:** 1 Department of Chemistry, University of Paderborn, Warburger Str. 100, D-33098 Paderborn, Germany; E-Mail: christian.weinberger@uni-paderborn.de; 2 Institute of Inorganic and Analytical Chemistry, Justus Liebig University, Heinrich-Buff-Ring 58, D-35392 Giessen, Germany; E-Mail: j-rocky@gmx.de; 3 Institute of Physics, University of Augsburg, Universitätsstraße 1, D-86135 Augsburg, Germany; E-Mail: jan.hanss@netzsch.com

**Keywords:** porous carbon, CMK-3, porous metal oxide, thermogravimetric analysis, mass ion detection, nanocasting, catalysis

## Abstract

A variety of metal nitrates were filled into the pores of an ordered mesoporous CMK-3 carbon matrix by solution-based impregnation. Thermal conversion of the metal nitrates into the respective metal oxides, and subsequent removal of the carbon matrix by thermal combustion, provides a versatile means to prepare mesoporous metal oxides (so-called nanocasting). This study aims to monitor the thermally induced processes by thermogravimetric analysis (TGA), coupled with mass ion detection (MS). The highly dispersed metal nitrates in the pores of the carbon matrix tend to react to the respective metal oxides at lower temperature than reported in the literature for pure, *i.e.*, carbon-free, metal nitrates. The subsequent thermal combustion of the CMK-3 carbon matrix also occurs at lower temperature, which is explained by a catalytic effect of the metal oxides present in the pores. This catalytic effect is particularly strong for oxides of redox active metals, such as transition group VII and VIII metals (Mn, Fe, Co, Ni), Cu, and Ce.

## 1. Introduction

Nanocasting is a well-established method of preparing ordered, mesoporous materials, such as mesoporous carbon, metals or metal oxides [1,2,3,4,5,6,7,8]. Typically, a mesoporous silica material serves as a structure matrix. Its pores are filled with a precursor compound for the desired product, e.g., sucrose [9] or other carbonic species [10,11] for porous carbon, or metal nitrates for metal oxides [5,6,7]. The precursor is then converted into the respective product inside the silica pores. The silica matrix is finally removed by selective chemical etching, either with hydrofluoric acid (HF) or with strongly alkaline solution (e.g., NaOH). The product can be envisaged as a “negative replica” of the silica matrix. However, many products, such as amphoteric metal oxides (e.g., Al_2_O_3_, ZnO, MgO, CuO, *etc.*), fail to withstand the etching procedure. In such cases, mesoporous carbon, e.g., CMK-3 [9], rather than silica, can be used as the structure matrix, as it can later be removed by thermal combustion upon slow heating under air. Since the carbon matrix is a negative replica prepared from a silica matrix, the final product may be regarded as a “positive replica” of the original parent silica material. It has recently been shown that this method is successful for the synthesis of mesoporous MgO [12,13,14], ZnO [15,16,17,18,19], CeO_2_ [14,20], Al_2_O_3_ [21,22,23,24], ZrO_2_ [21], TiO_2_ [21], and CuO [25]; further examples can be found in some related review articles [5,6,7,8]. We have also investigated the efficiency of impregnating porous carbon matrices with metal nitrates in some detail [26].

Here, were present the thermogravimetric analysis of the thermally induced processes during the nanocasting synthesis of various metal oxides by using mesoporous CMK-3 carbon as the structure matrix. Both the conversion of the respective metal nitrate into the metal oxide and the thermal combustion of the carbon matrix turn out to occur at temperatures different from those observed for the pure components.

## 2. Results and Discussion

**Figure 1:**
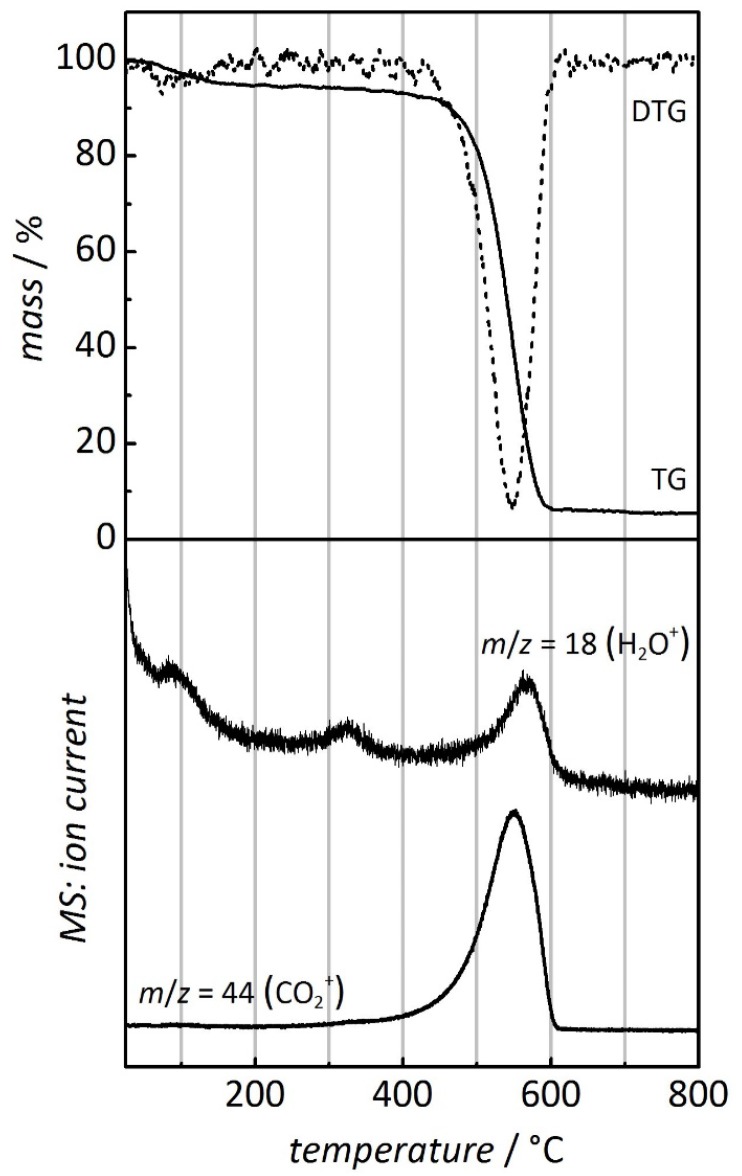
Thermogravimetric/derivative thermogravimetric (TG/DTG, (**top**)) and mass ion detection (MS, (**bottom**)) analysis of ordered mesoporous CMK-3 carbon. Heating rate: 10 °C min^−1^; atmosphere: O_2_/Ar, 20:80 vol.

Figure 1 shows the thermogravimetric analysis (TGA) of pure CMK-3 carbon that was prepared from sucrose by the standard literature procedures [9]. All thermal analyses was performed at a heating rate of 10 °C min^−1^. The gas atmosphere was O_2_/Ar (20:80 vol.) instead of air, in order to prevent interference of N_2_^+^ with CO^+^ in gas ion detection (as both ions exhibit the same mass). The thermal combustion of the CMK-3 carbon occurs at a temperature between *ca.* 450 °C and 610 °C. It is marked by a mass loss (TG signal) of *ca.* 90%, as depicted more clearly by the first derivative of the TG curve (DTG), accompanied by the detection of the CO_2_^+^ cation (*m/z* = 44). Small amounts of the H_2_O^+^ molecule cation (*m/z* = 18) are also observed during the combustion, which can be explained by some oxygen-containing organic functions in the CMK-3 carbon network, presumably at the pore wall surface. Prior to the combustion, especially in the temperature interval up to 100 °C, a mass loss of *ca.* 10% is observed which is attributable to the evaporation of physisorbed water, as confirmed by the detection of the H_2_O^+^ ion.

**Figure 2:**
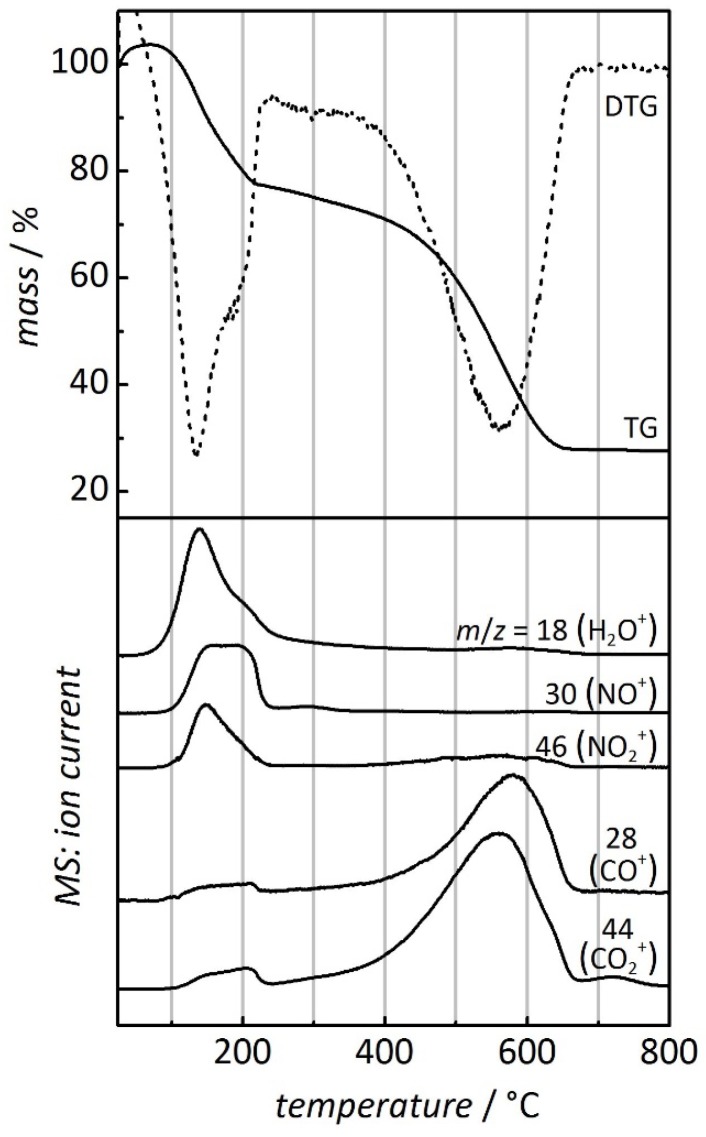
Thermogravimetric/derivative thermogravimetric (TG/DTG, (**top**)) and mass ion detection (MS, (**bottom**)) analysis of Al(NO_3_)_3_-impregnated ordered mesoporous CMK-3 carbon [20]. Please note that the cation with *m*/*z* = 46 may also correspond to ^12^C^16^O^18^O^+^, as stated in the text. Heating rate: 10 °C min^−1^; atmosphere: O_2_/Ar, 20:80 vol.

As stated in the introduction, mesoporous CMK-3 serves as a matrix for the synthesis of mesoporous metal oxides by structure replication (nanocasting). For this purpose an aqueous solution of a metal nitrate is typically filled into the carbon pores, followed by drying and subsequent conversion into the respective metal oxide by heating under air atmosphere. Depending on several factors, such as the metal nitrate’s solubility and the metal oxide’s density, this procedure typically leads to a filling of the carbon pores between 10% and 30% [2,3,4,5,6]. This procedure is applicable only if the temperature required for the conversion is lower than the temperature at which the CMK-3 carbon matrix will oxidize. This is usually the case, as will be shown below. Figure 2 displays the thermogravimetric analysis of ordered mesoporous CMK-3 carbon after infiltration of Al(NO_3_)_3_ as an example. Aluminum nitrate, like most other metal nitrates, my contain variable amounts of crystal water, depending on the drying conditions; in the following, we deliberately omit any such information, since the true amount of crystal water/residual solvent water is ill-defined. The thermal profile is marked by two distinct steps: (i) *ca.* 25% of the sample mass is lost between 100 and 255 °C. In this interval the NO^+^ (*m/z* = 30), NO_2_^+^ (*m/z* = 46), and H_2_O^+^ (*m/z* = 18) molecule ions are detected, confirming the conversion of Al(NO_3_)_3_ to Al_2_O_3_ and the release of water (presumably, mostly crystal water present in Al(NO_3_)_3_). Additionally, very weak signals of CO_2_^+^ (*m/z* = 44) and CO^+^ (*m/z* = 28) are observed at this temperature, which are absent in the thermal profile of pure CMK-3 carbon; apparently, the presence of finely dispersed Al(NO_3_)_3_ seems to have a weak oxidative/catalytic effect on the thermal combustion of carbon. (ii) The second step of mass loss (*ca.* 50%) occurs between 380 and 665 °C. This is attributable to the thermal combustion of the CMK-3 carbon, as confirmed by the detection of the CO_2_^+^ (*m/z* = 44) and the CO^+^ (*m/z* = 28) molecule cations. A weak signal is also observed in the detection of molecule cations with *m/z* = 46; this corresponds most likely to the ^12^C^16^O^18^O^+^ rather than the NO_2_^+^ cation. It is apparent that the temperature intervals in which the two instances (conversion of Al(NO_3_)_3_ to Al_2_O_3_ and carbon combustion) occur do not overlap. This has practical consequences in the nanocasting synthesis of Al_2_O_3_, as repeated cycles of nitrate infiltration and subsequent oxide formation turn out to be possible without premature damage of the CMK-3 carbon matrix. It may be worth mentioning that the final temperature of 800 °C may be higher than what is usually used in a true nanocasting synthesis. To preserve the nanoscopic structure and porosity, temperatures lower than 800 °C may be advantageous, in which case complete removal of the carbon matrix must be achieved by isothermic treatment for longer periods. For mesoporous Al_2_O_3_ we have previously used a maximum temperature of 450 °C with isothermic heating for 48 h [21].

Comparison of Figure 1 and Figure 2 reveals that in the Al(NO_3_)_3_-/Al_2_O_3_-loaded sample (i) the thermal combustion of carbon sets in at lower temperature, as will be discussed in detail below, and (ii) the release of CO_2_ lasts up to higher temperature. The latter observation may be attributable to adsorption of CO_2_ to the Al_2_O_3_ phase, potentially up to the point of formation of a carbonate species; the release of CO_2_ would then be delayed. Additionally, the gas-phase transport of oxygen through the pores of the carbon may be less effective owing to the presence of Al_2_O_3_; thus, the oxidation of carbon would be kinetically retarded and, therefore, completed at higher temperature under the non-isothermal conditions of this measurement.

**Figure 3:**
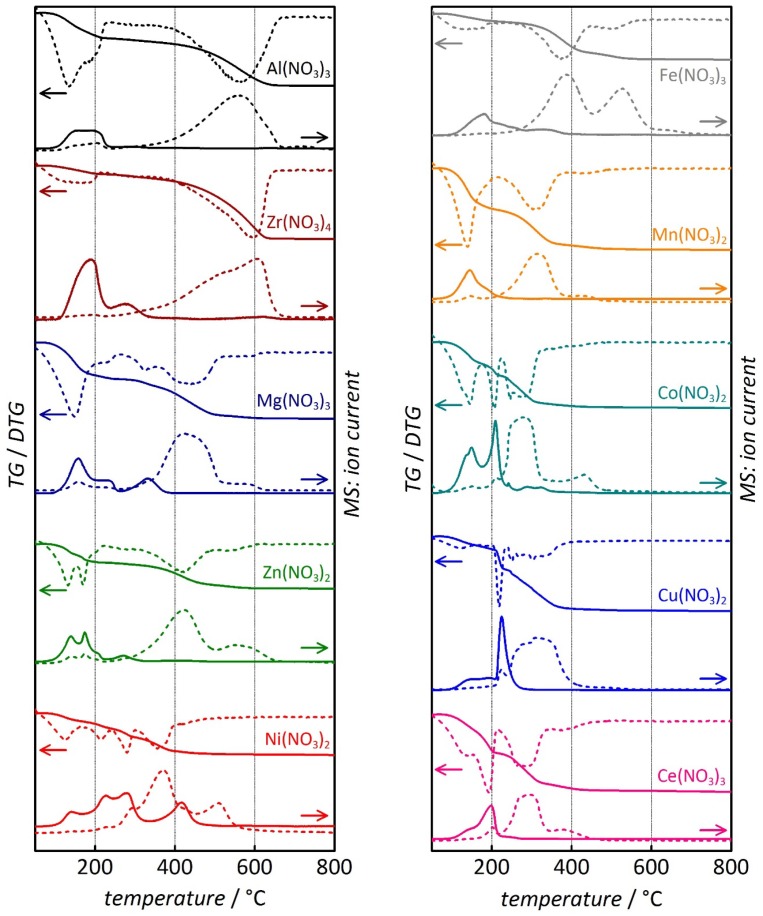
Thermogravimetric/derivative thermogravimetric (solid line TG/dashed line: DTG) and mass ion detection analysis (solid line: *m/z* = 30 (NO^+^)/dashed line: *m/z* = 44 (CO_2_^+^)) of various metal nitrate-impregnated ordered mesoporous CMK-3 carbon samples. Data for Mg(NO_3_)_2_ and Ce(NO_3_)_3_ previously shown in [13] and [20], respectively; heating rate: 10 °C min^−1^; atmosphere: O_2_/Ar, 20:80 vol.

**Figure 4:**
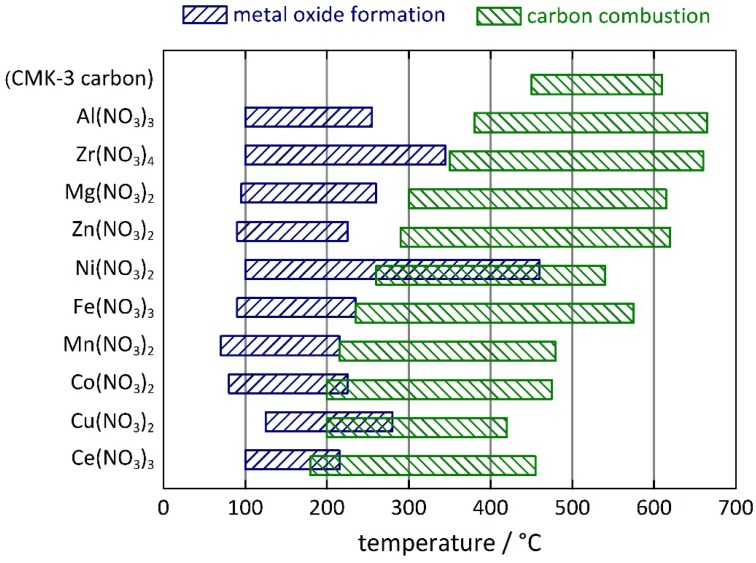
Comparing chart of the temperature intervals for the thermally induced formation of metal oxides from metal nitrates inside the pores of mesoporous CMK-3 carbon (blue bars) and for the thermal combustion of the carbon matrix (green bars).

The same analysis was performed for CMK-3 carbon loaded with several other metal nitrates in its pores, namely Zr(NO_3_)_4_, Mg(NO_3_)_2_ [13], Zn(NO_3_)_2_ [15], Ni(NO_3_)_2_, Fe(NO_3_)_3_, Mn(NO_3_)_2_, Co(NO_3_)_2_, Cu(NO_3_)_2_, and Ce(NO_3_)_3_ [20]. Figure 3 shows the mass loss (TG and derivative TG) as well as the signal of the NO^+^ (*m/z* = 30) and CO_2_^+^ (*m/z* = 44) molecule cation detection. In all cases, the thermal profiles resemble the same general picture as observed for the Al(NO_3_)_3_-impregnated sample in that two instances can be distinguished: (i) at relatively low temperature the conversion of the metal nitrate into the respective metal oxide occurs, as observed by a first step in the mass loss and by the detection of the NO^+^ cation (as well as the NO_2_^+^ and the H_2_O^+^ cations; not shown); (ii) At higher temperature the combustion of the porous CMK-3 carbon matrix occurs, as observed by a second step in the mass loss and by the detection of the CO_2_^+^ cation (as well as the CO^+^ cation; not shown). However, it is apparent that the respective temperature intervals, in which the two instances occur, differ from case to case, as depicted more clearly in Table 1 and Figure 4.

**Table 1:** Temperature intervals of the thermally induced conversion of metal nitrates into metal oxides in the pores of mesoporous CMK-3 carbon and of the thermal combustion of the carbon matrix.

Sample	Temperature (°C) of metal oxide formation			Temperature (°C) of carbon combustion	
	onset	offset	Literature *	onset	offset
CMK-3 carbon	-	-	-	450	610
Al(NO_3_)_3_	100	255	167	380	665
Zr(NO_3_)_4_	100	345	-	350	660
Mg(NO_3_)_2_	95	260	450	300	615
Zn(NO_3_)_2_	90	225	317	290	620
Ni(NO_3_)_2_	100	460	307	260	540
Fe(NO_3_)_3_	90	235	167	235	575
Mn(NO_3_)_2_	70	215	207	215	480
Co(NO_3_)_2_	80	225	242	200	475
Cu(NO_3_)_2_	125	280	247	200	420
Ce(NO_3_)_3_	100	215	297	180	455

Note: * temperature of thermal decomposition of metal nitrates according to [27].

The onset of the thermally induced, non-isothermic conversion of the metal nitrates into the respective metal oxides ranges from 70 to 125 °C. The temperature at which this conversion is finished varies more significantly, between 215 and 460 °C. The onset values are always lower than the decomposition temperatures for the pure metal nitrates (*i.e.*, in the absence of carbon) reported in the literature [27]; the latter were measured by thermal analysis with the same heating rate, but in air instead of Ar/O_2_ atmosphere. In some cases, even the offset temperature is lower than the literature value. Hence, it is fair to say that metal nitrates dispersed in the nanopores of CMK-3 carbon tend to decompose at lower temperature than otherwise. A possible explanation is the high dispersity of the metal nitrate in the carbon pores. In general, any comparison of thermal analysis data with previous ones needs to be carried out with great care; apart from the heating rate, thermal profiles may sensitively depend to a high degree on such experimental parameters as sample amount or crucible design.

More interestingly, the temperature interval for the thermal decomposition of the CMK-3 carbon matrix also varies substantially, depending on which metal nitrate is used. The onset temperature is always substantially lower than for the pure, *i.e.*, metal nitrate-free, CMK-3 carbon material (450 °C); is varies between 180 °C (for the Ce(NO_3_)_3_-loaded sample) and 380 °C (for the Al(NO_3_)_3_-loaded sample). The offset temperature follows the same trend; in most cases it is lower than for the pure CMK-3 carbon (610 °C). It is also worth noticing that the combustion of carbon rarely overlaps with the conversion of the metal nitrate into the respective metal oxide; such overlap is observed only for the three samples with the lowest onset temperature of carbon combustion (Co(NO_3_)_2_, Cu(NO_3_)_2_, and Ce(NO_3_)_3_), as well as for the Ni(NO_3_)_2_-loaded sample. The thermal behavior of the Ni(NO_3_)_2_-loaded sample is more complex than that of the other samples. Apparently, the reaction of (hydrous) Ni(NO_3_)_2_ to NiO occurs in several distinct steps, as also confirmed in the literature [28]. As a general outcome the data clearly show that the oxidative combustion of CMK-3 carbon is facilitated by the presence of the metal oxides that have formed inside the carbon pores. Obviously, the finely dispersed metal oxides catalyze the combustion, which results in lower combustion temperature. Moreover, this effect is correlated with the redox activity of the respective metal: a strong catalytic effect is observed for the oxides of transition group VII and VIII metals (Mn, Fe, Co, Ni), as well as of Cu and Ce. The oxides of less redox-active metals (Al, Mg, Zn, Zr) show weaker catalytic effect. Similar findings were observed for metal nitrate-impregnated activated carbon materials, although the effect was assigned to catalytic activity of the metal nitrates rather than the metal oxides [29]. Our data do not suggest that the metal nitrates themselves (instead of the metal oxides) have a predominant impact on the combustion of the carbon matrix, since in most cases the metal nitrates are completely converted to the respective metal oxides beforehand.

## 3. Experimental Section

### 3.1. Synthesis of SBA-15 Silica

Mesoporous SBA-15 silica (serving as the structure matrix for CMK-3 carbon) was synthesized by a standard procedure [30]. *Pluronic P123* triblock copolymer (16.00 g; Sigma-Aldrich, St. Louis, MS, USA) was dissolved in deionized water (480 mL) and hydrochloric acid (48 mL, 37%, Stockmeier, Bielefeld, Germany) und stirred overnight at 35 °C. After the addition of 32 mL tetraethyl orthosilicate (99%, abcr, Karlsruhe, Germany), the solution was stirred for 24 h at the same temperature. The dispersion was transferred into a glass-lined autoclave and hydrothermally treated at 140 °C for 24 h. After cooling down to room temperature the solid product was filtered off and washed with deionized water. After drying at 120 °C overnight the powder was treated in a tube furnace at 550 °C with a heating rate of 2.5 °C min^−1^ for 6 h to remove the polymer.

### 3.2. Synthesis of CMK-3 Carbon

Mesoporous CMK-3 carbon was synthesized by a modified standard procedure [9]. One gram of SBA-15 silica was mixed in 6 mL of an aqueous solution of 1.25 g sucrose (99.5%; Sigma-Aldrich) sulfuric acid (3%). The silica was added in small portions and stirred until a homogenous mixture was obtained. The mixture was treated in a drying cabinet at 100 °C for 6 h and again at 160 °C for the same time. The solid was ground and the procedure was repeated but with a reduced amount of sucrose (1.00 g). Finally, the resulting material was heated to 900 °C in a tube furnace under N_2_ flow for 4 h. To remove the silica matrix the composite material was treated with hydrofluoric acid two times, washed intensively with water and ethanol and dried at 120 °C overnight.

### 3.3. Impregnation of CMK-3 Carbon with Metal Nitrates

Mesoporous CMK-3 carbon was impregnated with metal nitrates (Al(NO_3_)_3_·9H_2_O, Sigma-Aldrich, 98%; Zr(NO_3_)_4_·5H_2_O, Sigma-Aldrich, 99%; Mg(NO_3_)_2_·6H_2_O, Merck, 99%; Zn(NO_3_)_2_·6H_2_O, Sigma-Aldrich, 98%; Ni(NO_3_)_2_·6H_2_O, Sigma-Aldrich, 97%; Fe(NO_3_)_3_·9H_2_O, Sigma-Aldrich, 98%; Mn(NO_3_)_2_·4H_2_O, Sigma-Aldrich, 99%; Co(NO_3_)_2_·6H_2_O, Sigma-Aldrich, 98%; Cu(NO_3_)_2_·3H_2_O, Sigma-Aldrich, 98%; Ce(NO_3_)_3_·6H_2_O, Merck, 98.5%) by the so-called incipient wetness method [5,6]. A saturated solution of the respective metal nitrate was mixed with the carbon material; the amount of solution was chosen in such a way as to match exactly the total pore volume (1.43 cm^3^ g^−1^, multiplied by the mass) of the carbon. The resulting paste was homogenized by grinding in a mortar and subsequently dried in vacuum at room temperature.

### 3.4. Thermal Analysis

Thermal analysis (TG/MS) was carried out in a Al_2_O_3_ crucible (*ca.* 10–25 mg sample mass) under O_2_/argon flow (20/80 volume ratio; flow rate: 50 cm^3^·min^−1^) with a Netzsch STA409PC thermobalance connected to a Balzers QMG421 quadrupole mass spectrometer by a custom-made coupling device. The heating rate was 10 °C min^−1^. Onset and offset temperatures were determined from the combined DTG curves and MS signals; any tailing of the respective signals was disregarded when its contribution to the overall peak was below 10%.

## 4. Conclusions

In summary, the thermally induced conversion of metal nitrates into the respective metal oxides inside the pores a mesoporous CMK-3 carbon matrix and the subsequent thermal combustion of the carbon matrix has previously shown to provide a versatile means of preparing mesoporous metal oxides. The processes can be monitored by thermogravimetry (TG), coupled with mass ion detection (MS) of the decomposition products (water, nitrogen oxides, carbon oxides). The decomposition of the metal nitrates dispersed in the porous carbon matrix turns out to occur at lower temperature than under carbon-free conditions. In most cases the combustion of the carbon matrix sets in only after the metal oxides have formed. However, the carbon combustion always takes place at lower temperature than in case of metal nitrate-/metal oxide-free CMK-3 carbon. This can be explained by a catalytic effect of the metal oxides, which is particularly strong for oxides of redox-active metals.

## Supplementary Materials

Supplementary materials can be accessed at: http://www.mdpi.com/2079-4991/5/3/1431/s1.
