# Supplementary material for: Synthesis of Mesoporous Metal Oxides by Structure Replication: Thermal Analysis of Metal Nitrates in Porous Carbon Matrices
Source: Nanomaterials (Basel). 2015 Aug 28;5(3):1431–41. doi: 10.3390/nano5031431 (PMC5304624; doi:10.3390/nano5031431)
Supplement: Supplementary file 1 [file nanomaterials-05-01431-s001.pdf]

Synthesis of Mesoporous Metal Oxides by Structure Replication: Thermal Analysis of Metal Nitrates in Porous Carbon Matrices

C. Weinberger, J. Roggenbuck, J. Hanss, M. Tiemann*

Supplementary Information

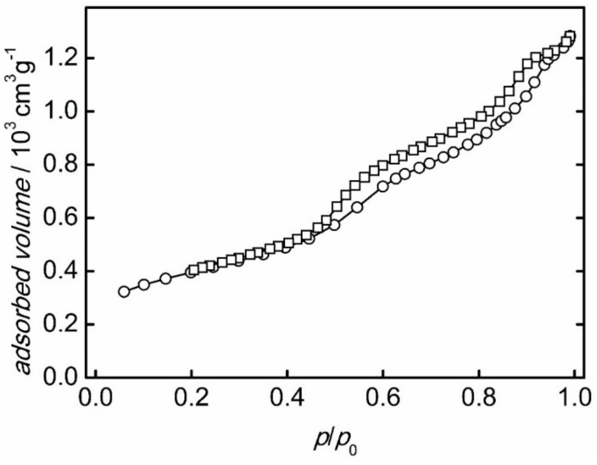

Figure S1. Nitrogen physisorption isotherm of ordered mesoporous CMK-3 carbon (circles, adsorption; squares desorption).

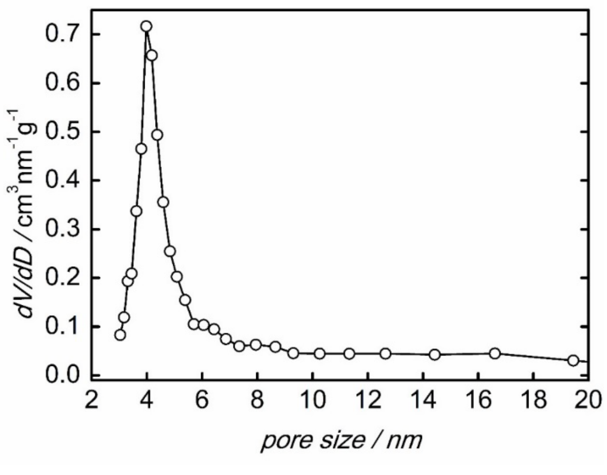

Figure S2 BJH-Pore size distribution (desorption branch) of ordered mesoporous CMK-3 carbon.

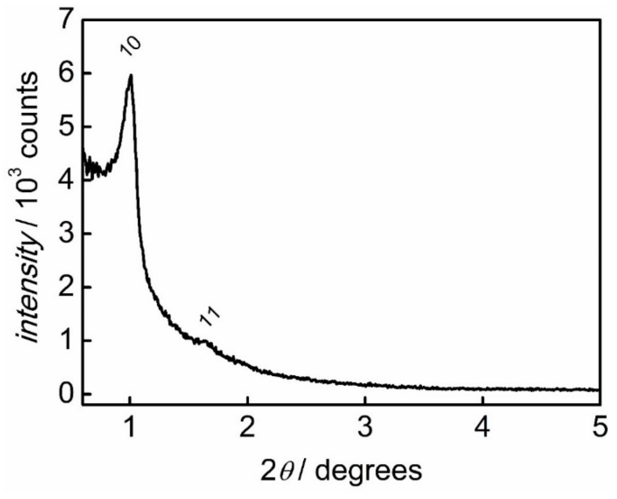

Figure S3. Powder X-ray diffraction pattern of ordered mesoporous CMK-3 carbon.

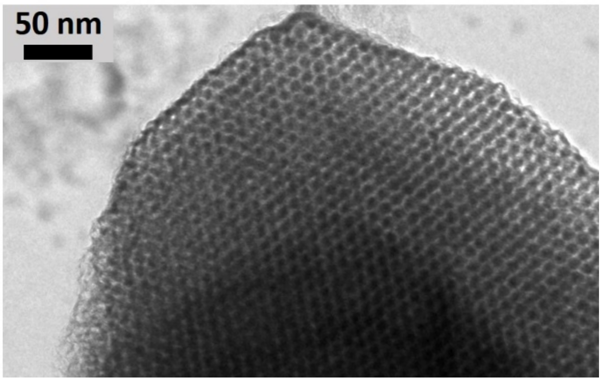

Figure S4. Representative transmission electron microscopy image of ordered mesoporous CMK-3 carbon.

Table S1. Structural parameters of mesoporous CMK-3 carbon.

BET surface area (m^2 g^{-1})	pore size (nm)	pore volume (cm^3 g^{-1})	lattice constant (nm)
1340	4.0	1.98	10.1
